# Third or fourth branchial pouch sinus lesions: a case series and management algorithm

**DOI:** 10.1186/s40463-019-0371-6

**Published:** 2019-11-11

**Authors:** Yun Li, Kexing Lyu, Yihui Wen, Yang Xu, Fanqin Wei, Haocheng Tang, Siyu Chen, Zhangfeng Wang, Xiaolin Zhu, Weiping Wen, Wenbin Lei

**Affiliations:** 1 Department of Otolaryngology, The First Affiliated Hospital of Sun Yat-sen University, Guangzhou, China; 2 Department of Otolaryngology-Head and Neck Surgery, Nanfang Hospital, Southern Medical University, Guangzhou, China

**Keywords:** Congenital branchial anatomies, Fistula tract excision, Pyriform sinus, Recurrent neck abscesses, Thyroiditis

## Abstract

**Background:**

The purpose of this study was to develop an effective management algorithm for lesions of third or fourth branchial sinuses.

**Study design:**

Case series with chart review.

**Methods:**

Data from patients who were identified as having third or fourth branchial pouch sinus lesions in a single institution between January 2014 and December 2018 were retrospectively collected.

**Results:**

All 67 patients underwent fistulectomy. First, we classified the patients into five types based on their anatomic features. Then, we considered four optimized surgical methods and adopted the appropriate method with full consideration of the patient’s clinical characteristics. The great majority of cases occurred on the left side of the neck (68.7%) and most commonly presented as either a recurrent low-neck abscess or cutaneous discharging fistula with neck infection. Effective preoperative examination included administering contrast agent prior to a computed tomography (CT) scan and in-office laryngoscopy during the quiescent period of inflammation. Ultrasound was also very helpful in determining the presence of thyroiditis. The mean follow-up duration after excision of the lesion was 25.8 months. To date, only 1 (1.5%) recurrence and no obvious complications have been observed.

**Conclusion:**

Refining fistula subtypes and adopting corresponding treatment measures can reduce the recurrence rate and improve curative effects. We propose and advocate this treatment algorithm for all third and fourth branchial pouch lesions.

Third or fourth branchial pouch anomalies are the most uncommon branchial anomalies, with a prevalence of 2 to 8% and 1 to 4%, respectively [1–3]. From the perspective of embryology, the third or fourth branchial pouch sinuses both originate at the pyriform sinus [4–6] and partially pass through or terminate in the upper thyroid lobes [7–9]. As a result of incomplete obliteration, lesions can clinically present as cysts, sinuses, or fistulas [10, 11]. Approximately 90% of these sinus tracts are situated on the left side of the neck, which may be due to embryonic development [12–14]. In the past twentieth century, according to most authors, the number of published cases was less than 100 [1, 11, 12, 15]. Recently, researchers have found that lesions in third or fourth branchial pouch sinuses are not uncommon. Nicoucar et al. [2, 3] found 526 cases, which is five times the previously identified prevalence, and the disease has gradually become a topic of debate in recent years.

The typical presentations of lesions in third or fourth branchial sinuses are recurrent low-neck abscesses, acute suppurative thyroiditis, and neck masses [2, 3, 11, 12, 15]. In the neonatal period, they usually present as a cystic mass or an abscess, which may lead to dyspnea with stridor, dysphagia, and feeding difficulties [16–18]. In the past, the only way to cure a third or fourth branchial fistula was to completely resect the affected tissue [12, 19]. Conventionally, this treatment consisted of surgical sinus tract excision with or without a hemithyroidectomy [2, 10, 11]. However, this surgery was challenging and resulted in large incisions with obvious postoperative scars. Complications and recurrence also occurred. Researchers have increasingly advocated for endoscopic cauterization of the sinus tract opening in the pyriform sinus [17, 20–22]. Unfortunately, this method will create a closed cavity. Moreover, relapse has been found to occur after secondary infection, especially for fistulas and inner sinus with thyroid abscesses. Therefore, it is challenging to treat lesions in third or fourth branchial sinuses.

In this study, we proposed a new treatment algorithm for treating lesions in third or fourth branchial sinuses on the basis of our theoretical and practical experience. First, we advocated refine clinical subtypes based on clinical features and fistula shape. Then, we modified the surgical approach that was used to identify and excise the tract. Finally, accurate disease classification and diagnosis were achieved, allowing personalized treatment plans to be implemented. From our data, we found that this approach could improve treatment effects, reduce trauma and significantly reduce the recurrence rate (to 1.5%).

## Materials and methods

### Data extraction

We retrospectively reviewed 67 cases of third or fourth branchial pouch sinus lesions treated in the Otolaryngology Department of the First Affiliated Hospital of Sun Yat-sen University, China, from January 2014 to December 2018. Sex, side of presentation, age at onset, age at diagnosis, mode of presentation, diagnostic investigations, treatment, operative details and postoperative course were noted from the case records. Complications and recurrence were also recorded at follow up. Diagnoses were made based on clinical manifestation, symptoms, preoperative radiological demonstration of a fistulous tract [using contrast-medium swallow and computed tomography (CT) fistulography], intraoperative findings, and histopathological analysis. Fistulectomy was performed in all cases.

### Ethical considerations

This study was approved by the Sun Yat-sen University Ethics Committee for Research and Publication.

### Principle of treatment (Fig. 1)

During the inflammatory infection period, antibiotics were administered in response to bacteriologic cultures. In the early period, we used β-lactam antibiotics that are active against streptococci and staphylococci. If the antibiotics were ineffective, the patient was given a puncture or underwent incision drainage. During the quiescent period of inflammation, we selected the appropriate treatment among the following four surgical methods according to the specific classification of the third or fourth branchial pouch lesion.

- Type I Initial outer sinus or outer sinus with cysts or isolated cysts

**Fig. 1:**
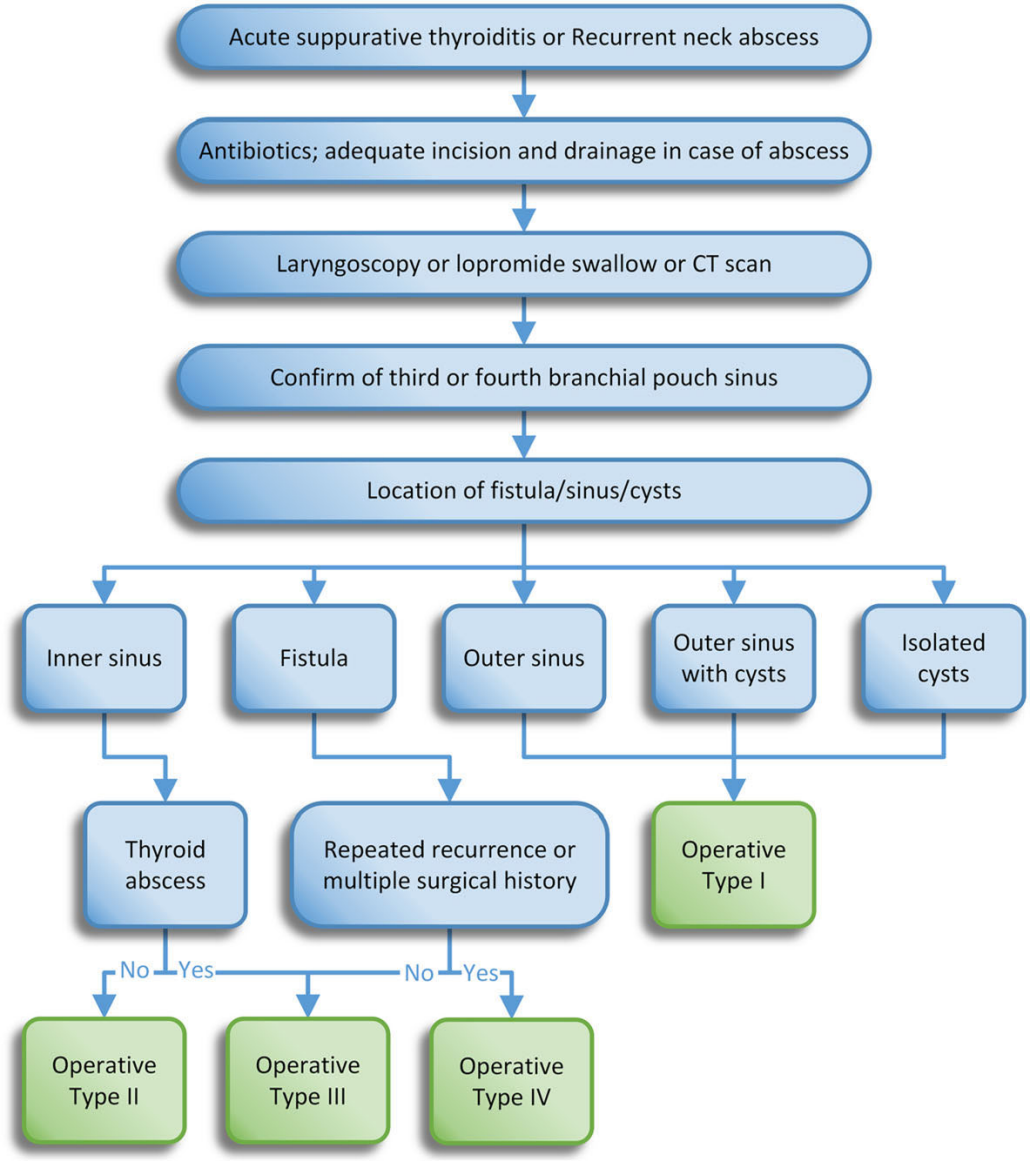
Suggested algorithm for the management of lesions in third or fourth branchial pouch *sinuses.* (Operative type I: traditional open surgery; Operative type II: internal fistula resection by CO_2_ laser under a suspension laryngoscope and endoscope; Operative type III: internal fistula resection by CO_2_ laser combined with external fistula resection by small neck incision; Operative type IV: complete resection of the fistula, assisted by the lumbar anesthesia tube as a metal probe combined with a pattern of neck dissection)

From the path along the neck skin lines, we dissected the lesion from the scar and the normal tissue boundary (if the lesion is a cyst, we dissected from the middle of the swollen object). Preoperatively, we injected methylene blue along the external leak to distinguish the sinus from the surrounding normal tissue. Then, we gradually dissected the fistula starting before the bifurcation of the common carotid artery. The separated fistula was extracted and the enlarged lymph nodes around the vascular sheath were cleared. If the adhesion between a slim fistula and granulation scar tissue was difficult to identify, we performed a functional neck dissection.

(b) Type II Initial inner sinus

**Fig. 2:**
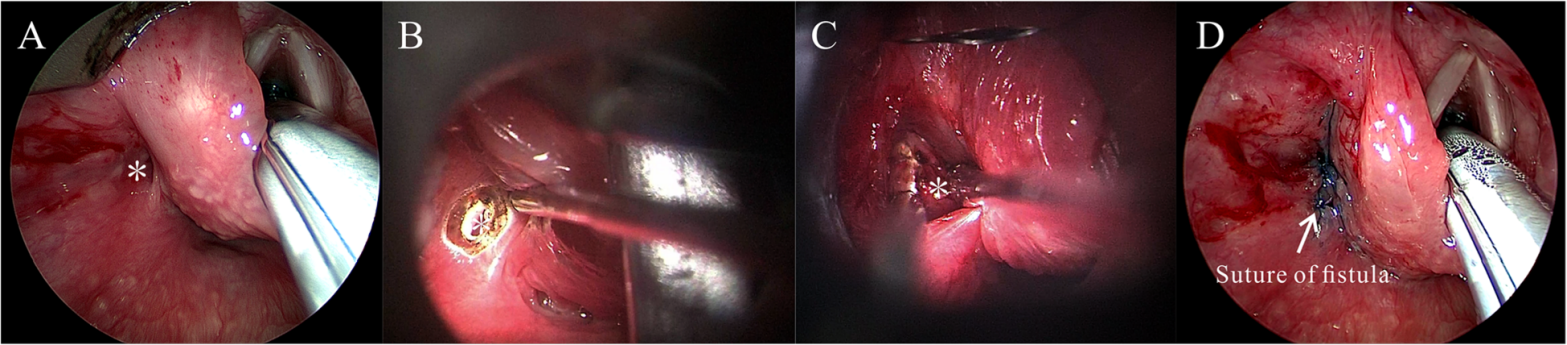
Internal fistula resection by CO_2_ laser under a suspension laryngoscope and *endoscope.* (*: internal fistula)***.*** **a** Exposing the fistula with assistance from the suspension laryngoscope and endoscope;. **b** Making a circular incision around the mouth of the fistula with the CO_2_ laser;. **c** Excising and separating the fistula along the tissue space outside the fistula;. **d** Closing the incision with 7–0/8–0 absorbable suture

Intraoperatively, we applied a suspension laryngoscope and endoscope to fully expose the fistula in the affected side of the piriform fossa, Betz mucosal folds (i.e., longitudinal mucosal folds at the inside of the piriform fossa and at the entrance to the esophagus), and esophageal entrance. Under the operating microscope, we used a CO_2_ laser to make a circular incision around the mouth of the fistula. Then, we dissected the fistula by excising and separating the tissue space outside the fistula, which extended straight back to the thyroid cartilage lamina and was usually (89.5% of cases) located behind the thyroid cartilage. Finally, the few residual fistula tissues were directly cut and vaporized by the laser, after which the incision was closed with 7–0/8–0 absorbable suture (Fig. 2, Additional file 3**: Video S1**).

(c) Type III Inner sinus combined with thyroid abscess and nonrefractory fistula

**Fig. 3:**
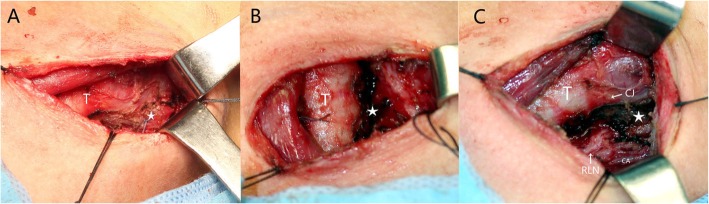
Small incision to excise the external fistula. **a** Making a small incision along the neck skin (T: trachea, ★: external fistula). **b** Dissecting to the CJ (cricothyroid joint) along the scar and normal tissue gap. **c** Dissecting around the important anatomical features, namely, the recurrent laryngeal nerve (RLN) and carotid artery (CA), and completely removing the entire lesion of the fistula, the surrounding scar and the involved upper lobe of the thyroid

Intraoperatively, we applied a suspension laryngoscope and endoscope to fully expose the fistula on the affected side of the piriform fossa. Under the operating microscope, we used the CO_2_ laser to excise the piriform fossa fistula and then closed the incision with 7–0/8–0 absorbable suture. Next, we made a small incision along the neck skin (Fig. 3a). For patients who had little inflammatory scar tissue in the neck and no obvious inflammation in the thyroid gland, we found the fistula usually directly along the normal tissue space around the cricothyroid joint and then removed it together with the surrounding scar without dissection of the recurrent laryngeal nerve. For patients who also had combined ipsilateral thyroiditis, we tried to keep the thyroid membrane intact and protect the recurrent laryngeal nerve. Therefore, we dissected the posterior border of the thyroid cartilage along the scar and normal tissue gap. Then, we removed the entire fistula as well as the surrounding scar and the involved upper lobe of the thyroid (Fig. 3b-c). If necessary, we also removed the lower corner of the thyroid cartilage and additional thyroid cartilage to fully expose the fistula, thereby facilitating its removal. Finally, we used pouch inversion stitching to correct the piriform fossa defect.

(d) Type IV Refractory fistula with repeated recurrence or a history of multiple surgeries

**Fig. 4:**
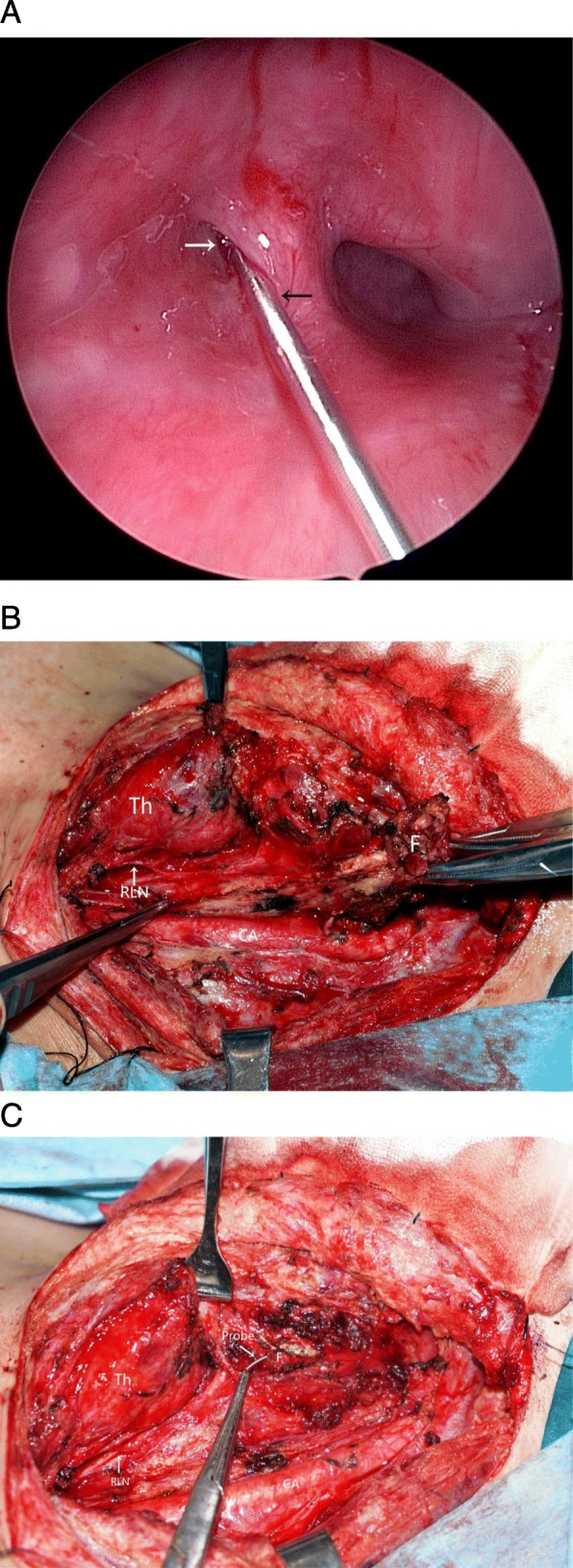
Complete resection of the fistula with the assistance of a probe combined with a pattern of neck dissection*.* **a** Inserting the probe into the internal opening of the branchial pouch sinus; (white arrow: tract opening; black arrow: metal probe). **b** Dissecting around the important anatomical features. (RLN: recurrent laryngeal nerve; CA: carotid artery; Th: thyroid; F: fistula). **c** Tracing the inner fistula where the probe is positioned and completely removing the fistula and surrounding scar. (RLN: recurrent laryngeal nerve; CA: carotid artery; Th: thyroid; F: fistula)

In the group that suffered from recurrent neck infection or abscesses, most patients had a history of multiple surgeries. As a result, the fistula was typically surrounded by scar tissue and adhesions, and important anatomical structures were not clearly observable, which led to difficulties in isolating and removing the fistula **(**Additional file 1: Figure S1). Intraoperatively, we used a probe to explore the internal opening of the pyriform sinus fistula and guide the fistula entrance to the larynx to affix it to the hypopharyngeal wall (Fig. 4a). We then dissected along the scar and normal tissue gap. First, we dissected the recurrent laryngeal nerve and internal carotid artery (Fig. 4b). Second, we traced along the gap between the scar tissue and the normal tissue to the inner fistula where the probe was positioned and then completely removed the fistula and the surrounding scar tissue (Fig. 4c) If the thyroid was involved, the inflamed thyroid tissue was removed as well. Finally, a purse string suture was used to ligate the piriform fossa to complete the fistula resection.

## Results

Sixty-seven cases of third or fourth branchial lesions were included during the study period (fistulas = 25 patients, inner sinus = 16 patients, outer sinus = 18 patients, outer sinus with cysts = 1 patient, isolated cysts = 7 patients). To date, only 1 (1.5%) recurrence and 2 (3%) patients with wound infections have been observed. The demographic and clinical characteristics of the five groups of patients are separately presented in Table 1.

**Table 1:** Features of five groups of third/fourth branchial arch anomalies

	I	II	III	IV	V
Cases (67 cases) [n (%)]	25 (37.3%)	16 (23.9%)	18 (26.9%)	1 (1.5%)	7 (10.4%)
Demographic					
Female [n (%)]	16 (64.0%)	9 (56.3%)	8 (44.4%)	1 (100.0%)	6 (85.7%)
Age [median (range)]	9y(1y-47y)	8y(1y-35y)	11.5y(1y-39y)	16y(16y)	21y(7y-42y)
Site of presentation (n [%])					
Left	22 (88.0%)	14 (87.5%)	8 (44.4%)	0 (0.0%)	2 (28.6%)
Right	0 (0.0%)	1 (6.3%)	7 (38.9%)	1 (100.0%)	5 (71.4%)
Bilateral	3 (12.0%)	1 (6.3%)	3 (16.7%)	0 (0%)	0 (0%)
Initial presentation (n [%])					
Recurrent low-neck abscess	24 (96.0%)	14 (87.5%)	2 (11.1%)	1 (100%)	1 (14.3%)
Acute suppurative thyroiditis	16 (64.0%)	4 (25.0%)	0 (0.0%)	0 (0.0%)	0 (0.0%)
Neck mass	1 (4.0%)	2 (12.5%)	7 (38.9%)	0 (0.0%)	6 (85.7%)
Cutaneous discharging fistula with neck infection	11 (44.0%)	4 (25.1%)	9 (50.0%)	0 (0.0%)	2 (28.6%)
Diagnostic investigations performed (n [%])					
Iopromide swallow (41 cases)	15 (60.0%)	6 (87.5%)	/	/	/
Laryngoscopy (41 cases)	21 (84.0%)	10 (62.5%)	/	/	/
CT scan (51 cases)	22 (88.0%)	14 (87.5%)	8 (44.4%)	1 (100.0%)	4 (57.1%)
Ultrasound (31 cases)	13 (92.9%)	9 (90.0%)	4 (100.0%)	1 (100.0%)	2 (100.0%)
MRI (13 cases)	3 (100.0%)	4 (100.0%)	1 (50.0%)	1 (100.0%)	3 (100.0%)
Treatment (n [%])					
Incision and drainage	22 (88.0%)	7 (43.8%)	6 (33.3%)	1 (100.0%)	1 (14.3%)
Open neck surgery after failure	10 (40.0%)	0 (0.0%)	1 (5.6%)	0 (0.0%)	0 (0.0%)
None	3 (12.0%)	9 (56.2%)	9 (61.1%)	0 (0.0%)	6 (85.7%)
Operating method (n [%])					
Type I	/	/	18 (100.0%)	1 (100.0%)	7 (100.0%)
Type II	/	10 (62.5%)	/	/	/
Type III	16 (64.0%)	5 (31.3%)	/	/	/
Type IV	9 (36.0%)	1 (6.3%)	/	/	/
Complication (n [%])					
Wound infection	0 (0%)	2 (12.5%)	0 (0%)	0 (0%)	0 (0%)
Esophageal injury	0 (0%)	0 (0%)	0 (0%)	0 (0%)	0 (0%)
Voice hoarseness	0 (0%)	0 (0%)	0 (0%)	0 (0%)	0 (0%)
Recurrence (n [%])	0 (0%)	1 (6.25%)	0 (0%)	0 (0%)	0 (0%)

Group I: Fistulas; Group II: Inner sinus; Group III:Outer sinus; Group IV:Outer sinus with cysts; Group V:Isolated cysts

Most cases were diagnosed in childhood, and most of these patients endured multiple incisions for drainage or fistula resection before the establishment of a clear diagnosis. The great majority of cases occurred on the left side of the neck (68.7%), and the most common presentation was either a recurrent low-neck abscess or cutaneous discharging fistula with neck infection (Table 1). The numbers of men and women were similar among the included patients (Table 1).

### Diagnostic investigations

The examination date data showed the specific examinations that were performed on each patient to reveal the branchial lesions. The techniques usedInvestigations used (and their positive predictive values [PPV’s]) were contrast-medium swallow in 61.2% of cases (PPV, 51.2%), in-office laryngoscopy in 61.2% (PPV, 75.6%), magnetic resonance imaging (MRI) in 19.4% (PPV, 92.3%), and computed tomography (CT) in 76.1% (PPV, 94.1%). The sensitivity of thyroid abscess detected detection by ultrasound thyroid scan was 93.5%. All the results were confirmed during the surgical procedure. Notably, in routine practice, we prefer to use iopromide instead of barium for contrast esophagography because barium has residual effects that can affect the surgeon’s ability to determine the shape of the fistula.

### Operative findings

Among the 67 cases, 25 were fistulas (of which 9 cases had a history of multiple surgeries), 16 were inner sinus lesions (of which 5 cases presented with thyroid abscesses), 18 were outer sinus lesions, 1 was an outer sinus lesion with cysts, and 7 were isolated cysts. Only 10 patients underwent CO2 laser ablation of the fistula under a suspension laryngoscope and endoscope, while 57 patients underwent open surgical resection of the fistula. Of the 41 patients who had an internal fistula, 20 cases (48.8%) involved fistulas that passed directly through the thyroid gland, and 21 cases (51.2%) involved fistulas that terminated in the upper lobe of the thyroid gland. Of the patients who had combined thyroiditis and ineffective antibiotic treatment, we removed the involved upper lobe of the thyroid along with the fistula. Of the 25 patients who had fistulas, we found that the fistula of one patient penetrated through the thyroid cartilage, while the others exited the piriform fossa and bypassed the posterior border of the thyroid cartilage before piercing through the inferior constrictor. Interestingly, 3 patients with unilateral neck swelling underwent intraoperative laryngoscopy, which revealed that both sides of the piriform fossa had a fistula. All 3 patients were treated with fistulectomy. None of the fistula tracts looped around the hypoglossal nerve or carotid arteries, and none descended into the mediastinum.

### Modified procedures

For patients with inner sinus lesions combined with thyroid abscesses and nonrefractory fistulas, we primarily used a CO2 laser to resect the internal fistula. Then, we excised the external fistula from the neck through a small incision and also removed the surrounding scars in the upper lobe of the involved thyroid via this incision. Compared with conventional surgical methods, this operation did not require dissection of the recurrent laryngeal nerve (if it was not combined with thyroiditis) and was simpler, with a smaller incision and less neck scarring (Additional file 2: Figure S2). Notably, previous scarring on the neck could also be removed.

### Outcomes

All 67 patients underwent fistulectomy by the appropriate surgical approach according to their clinical features and fistula shape, and the in-office laryngoscopy and esophagography results were reviewed every 3 months after surgery. The mean follow-up duration after excision of the lesion was 25.8 months (6 months–56 months). Table 2 presents the recurrence rates and complications during the study period. Compared with the previous recurrence rate (15.2%), which was recorded by Nicoucar et al. [2, 3] from an analysis of published cases, only one patient exhibited recurrence, and the recurrence rate in our study after clinical subtype refinement was 1.5% (*p* = 0.002). The patient who had recurrence was in the initial inner sinus group and was treated using a CO2 laser under a suspension laryngoscope and endoscope. On recurrence, we found that the patient had a fistula that passed through the thyroid gland, and we performed the surgery again combined with excision of the external fistula from the neck through a small incision. Subsequently, we strengthened the evaluation of the fistula shape before surgery, and there were no more instances of recurrence. Two wound infections occurred postoperatively; the affected patients were also in the initial inner sinus group, and the infections disappeared after treatment with a sufficient course of antibiotics. The occurrence of postoperative infection was greatly reduced after strengthening the perioperative evaluation of the patient’s inflammation. To date, no other postoperative complications, such as salivary fistula, vocal cord paralysis or arytenoid edema, have occurred.

**Table 2:** Results of post-grouping and ungrouped patients

Clinical presentation	ungrouped	After grouping	P
Recurrence	15.2%(67/439)	1.5%(1/67)	0.002^*^
Complication			
Salivary fistula	0.9% (4/439)	0% (0/67)	0.433
Infected wound	0.2% (1/439)	3% (2/67)	0.006
Vocal cord paralysis	3.2% (14/439)	0% (0/67)	0.138
Arytenoids edema	0.2% (1/439)	0% (0/67)	0.696

## Discussion

Third branchial arch tract anomalies originate from the base of the piriform fossa, while fourth branchial arch tract anomalies originate from the apex of the piriform fossa. Both types mostly pass through the throat near the cricothyroid joint, and their external fistula orifice is open to the skin of the anterior border of the sternocleidomastoid muscle. Because it is rather difficult to distinguish the lesions that develop from third and fourth branchial pouch anomalies, some researchers treat them as the same disease [2, 3, 10, 11].

Third and fourth branchial pouch sinuses are complex, and the corresponding lesions can occur anywhere in the fistula course. Depending on the presence of internal or external fistulas, third and fourth branchial pouch sinus lesions are divided into three types: sinus, fistula, and cyst types, which have been previously described in the literature [1–3, 10, 23, 24]. Among them, the sinus type is the most common. The fistula type is generally caused by secondary infection, abscess ulceration, or repeated iatrogenic incision and drainage. Because cases with repeated drainage incisions or multiple recurrence events after surgery were referred to our hospital, the proportion of the fistula type (37.3%) was higher in our study. Based on the observation of a large number of cases and consideration of medical history, physical signs, imaging examinations, and in-office laryngoscopy, we advocate clinically refined subtypes for patients who have third or fourth branchial pouch sinus lesions, which could provide a more effective treatment strategy.

This study included 20 patients with thyroid abscesses, 16 of which had an inner sinus. According to Nicoucar et al. [2, 3], among 62 patients receiving purely endoscopic procedures, the failure rate after the initial procedure was 15% (9 patients). At the start of this study, internal fistula resection was conducted in patients with an inner sinus using a CO_2_ laser under a suspension laryngoscope and endoscope after inflammation was controlled. However, we found that in a patient with a thyroid abscess who only underwent internal fistulectomy with a CO2 laser, the neck abscess reappeared on the fifth day after surgery. This reappearance might have occurred because inflammation is difficult to completely eliminate by administering antibiotics alone, particularly in the thyroid, which is covered by thick fibers. Furthermore, during surgery, we found that patients with thyroid abscesses had fistulas that always passed through the thyroid gland. Therefore, for patients with a fistula and thyroid abscess, complete removal of the fistula was critical. On this basis, we advocate a new surgical procedure to treat such patients. We primarily used a CO2 laser to resect the internal fistula and then completely removed the external fistula along the skin through a small incision in the neck. The operation was simple and shorter in duration than previous approaches. Additionally, we observed less trauma, fewer complications, and fewer scars in our patients. In the following 13 patients with inner sinus and thyroid abscess, no recurrence was found after the procedure. For patients with a fistula that has not been surgically resected (excluding incision and drainage), we also advocate using this procedure to reduce the number of residual epithelial cells as much as possible, thereby minimizing recurrence. To date, no recurrence has been observed.

Accurately classifying and diagnosing disease is necessary to develop effective treatment plans. Achieving a systematic and integrated treatment path is our common pursuit. In the past, Goff CJ et al. [25] proposed a treatment algorithm for branchial fistula, while Li YX et al. [26] introduced the methods of surgical treatment in patients with third and fourth branchial anomalies, but we consider these existing approaches to be insufficient. Combining our theoretical and practical experience, we advocate for refinement of the clinical subtypes in patients with third or fourth branchial pouch sinus lesions to optimize diagnoses and provide individualized and precise treatment. Because the positive diagnosis rate of barium esophagoscopy and CT scanning is low and fistulas are often small, radiologists often miss key findings or misdiagnose patients. In this study, we found that asking patients to take contrast agents prior to the CT scan during the quiescent period of inflammation and reminding surgeons to perform readings before surgery could help them identify fistulas, clarify the relationship between the fistula and adjacent structures and further aid classification. In addition, lifting the patient’s neck skin and asking patients to cheek blow under in-office laryngoscopy can better expose the tip of the piriform fossa, which aids in detection of an internal fistula and reduces the rate of missed diagnosis. By the way, the in-office laryngoscopy is an in-office, digital, lighted flexible laryngoscopy. Among the patients with an internal fistula, the positive rate was as high as 75.6%. In addition, ultrasound is also very helpful in diagnosis, especially in determining the presence of thyroiditis. Under normal conditions, thyroid tissue has a rich blood supply and a thick fibrous envelope that protects against infection [7, 8, 27, 28]. Therefore, when patients present with repeated neck abscesses and suppurative thyroiditis – especially on the left side – this disease should be considered. Through comprehensive examination, we can significantly reduce the rate of missed diagnoses and combined with assessment of the corresponding clinical features achieve individualized diagnosis.

For patients with different clinical classifications, we adopted corresponding methods to provide individualized treatment. For patients who experienced recurrence after laser surgery, it was difficult to determine the position of the internal fistula because of iatrogenic closure of the internal fistula. We decided to re-evaluate these patient according to the preoperative neck enhancement CT and medical history before deciding which surgical procedure to use. We also continuously improved the surgical procedure used on patients. For patients with an internal fistula requiring open surgery, we placed a gelatin sponge or Naxi cotton with staining agent through the internal fistula under suspension laryngoscopy and endoscopy to provide a marker for identifying the fistula during open surgery. Because of its absorbability, it was not necessary to remove the marker with the suspension laryngoscope or endoscope after surgical removal of the fistula. This operation method was simpler, had a shorter operative duration, and resulted in less damage.

Our data showed that there were no complications, such as permanent recurrent laryngeal nerve injury or hypothyroidism, and the recurrence rate (1.5%) was significantly reduced. After adopting the corresponding treatment measures according to clinical classification of the third or fourth branchial pouch sinus lesions, we significantly improved the outcomes. Therefore, it is possible to use clinical classification to provide an effective treatment algorithm. A prospective study would help clarify uncertainties but is not plausible given the rarity of this disease.

## Conclusion

Third or fourth branchial pouch sinus lesions can be characterized as congenital cervical cysts, sinuses, or fistulas. Refining the different fistula subtypes and adopting the appropriate treatment according to specific classification of third and fourth branchial pouch lesions can greatly reduce the recurrence rate and improve outcomes. We advocate this approach for all third or fourth branchial pouch sinus lesions.

## Supplementary information

**Additional file 1: Figure S1.** Patient who had a refractory fistula with repeated recurrence and a history of multiple surgeries.

**Additional file 2: Figure S2.** Patient with a smaller incision and less neck scarring 7 days after operation.

**Additional file 3: Video S1.** Operative type II: Internal fistula resection by CO_2_ laser under suspension laryngoscopy and endoscopy.

## Data Availability

All data are available upon request from the authors.
