# Supplementary figures and images for: Third or fourth branchial pouch sinus lesions: a case series and management algorithm
Source: J Otolaryngol Head Neck Surg. 2019 Nov 11;48:61. doi: 10.1186/s40463-019-0371-6 (PMC6849311; doi:10.1186/s40463-019-0371-6)

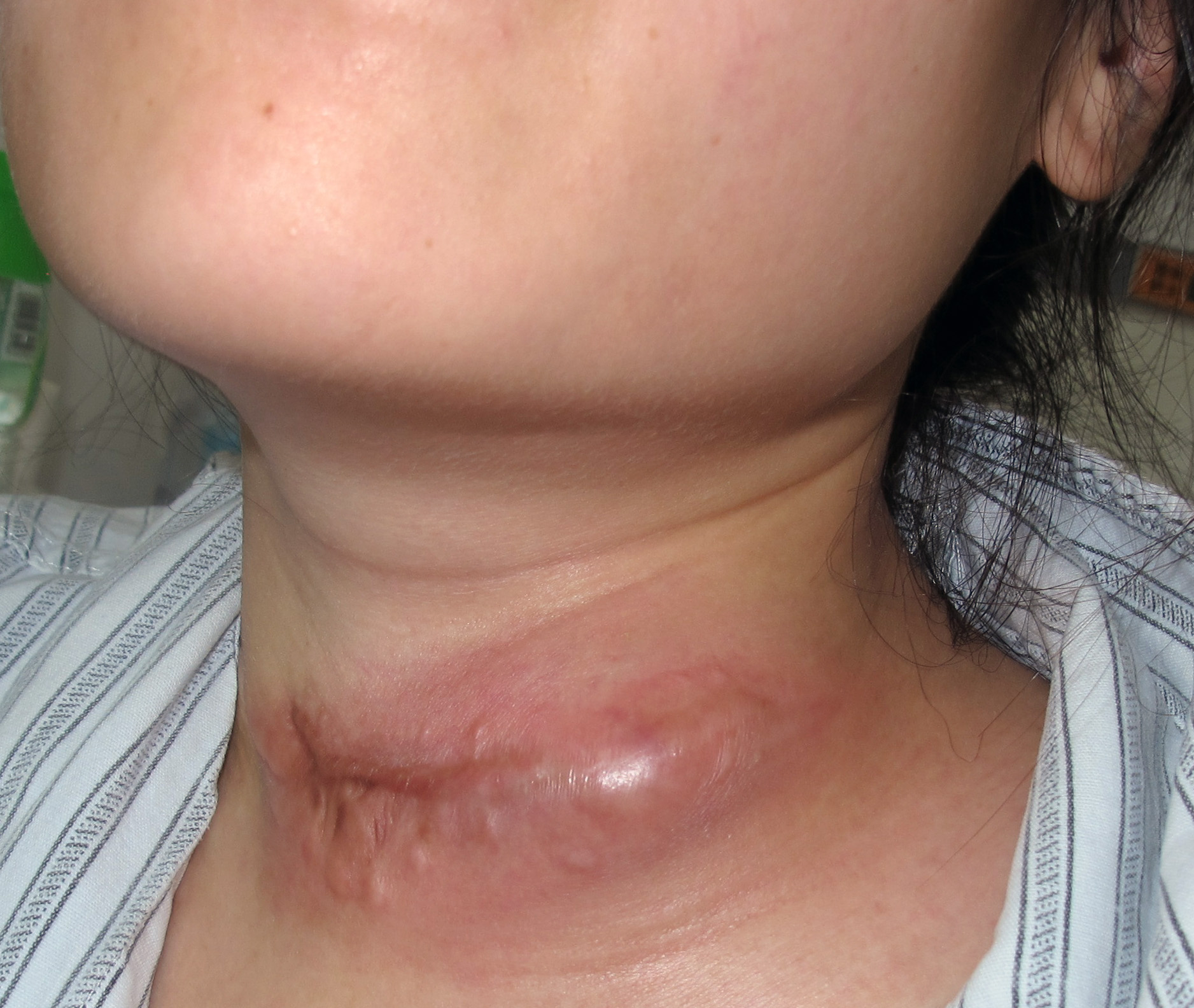

Supplement: Supplementary file 1 — Additional file 1: Figure S1. Patient who had a refractory fistula with repeated recurrence and a history of multiple surgeries. [file 40463_2019_371_MOESM1_ESM.jpg]

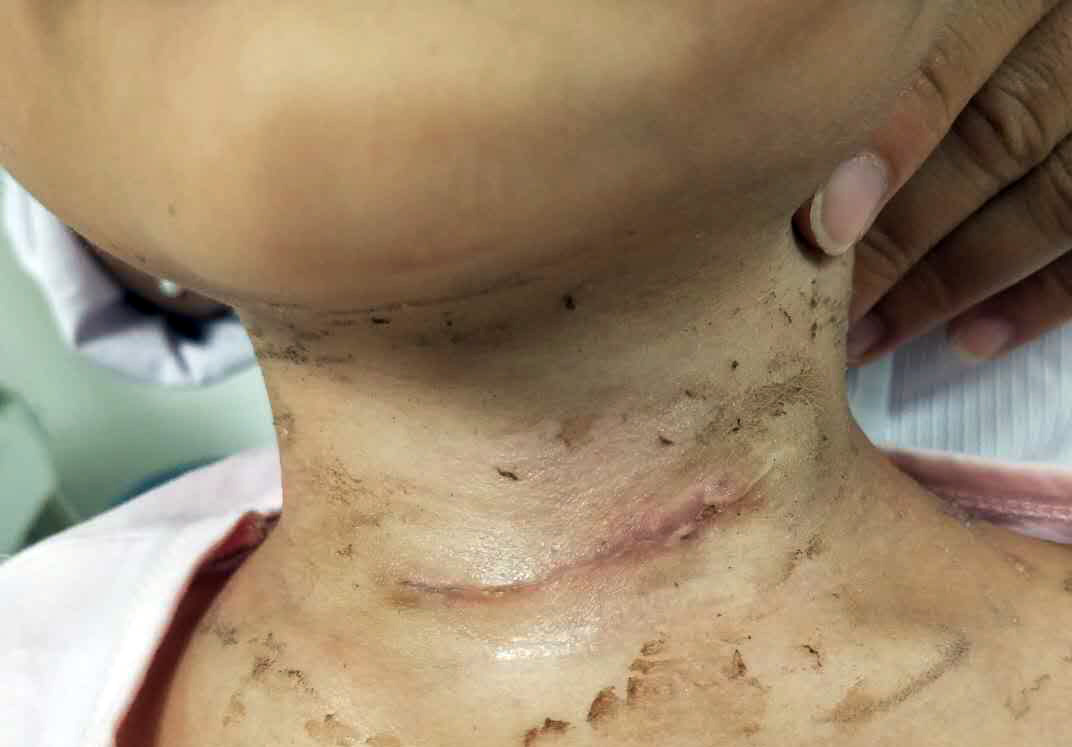

Supplement: Supplementary file 2 — Additional file 2: Figure S2. Patient with a smaller incision and less neck scarring 7 days after operation. [file 40463_2019_371_MOESM2_ESM.jpg]
